# AI Models for Suicide Risk Prediction in Adult Patients Receiving Mental Health Care Using Real-World Data: Retrospective Population-Based Study

**DOI:** 10.2196/85085

**Published:** 2026-08-26

**Authors:** Cleofé Peña-Gómez, Marc Fradera, Xavier Sánchez Corrales, Marc Caravaca-Rodriguez, Juan-Francisco Martínez-Cerdá, Joan Albert Escofet, David Roche, Eneko Barbería, Caridad Pontes, Jesús Giraldo, Diego Palao

**Affiliations:** 1 Barcelona Supercomputing Center, Barcelona, Spain; 2 Institut d’Investigació i Innovació Parc Taulí (I3PT-CERCA), Plaça Torre de l'Aigua, s/n,, Sabadell, 08208, Spain, +34 937458376; 3 Unitat Mixta de Neurociències Traslacional I3PT-INc-UAB, Universitat Autònoma de Barcelona, 08208 Sabadell, Spain; 4 Centro de Investigación Biomédica en Red de Salud Mental (CIBERSAM), Instituto de Salud Carlos III, 28029 Madrid, Spain; 5 Department of Mental Health, University Hospital Parc Taulí, 08208 Sabadell, Spain; 6 Agency for Health Quality and Assessment of Catalonia (AQUAS), Data Analytics Program for Health Research and Innovation (PADRIS) of the Data and AI Department, Barcelona, Spain; 7 Research Institute for Evaluation and Public Policies (IRAPP), Universitat Internacional de Catalunya (UIC), Barcelona, Spain; 8 Forensic Pathology Department, Institut de Medicina Legal i Ciències Forenses de Catalunya (IMLCFC), Barcelona, Spain; 9 School of Medicine and Health Sciences. Universitat Rovira i Virgili (URV), Reus, Spain; 10 Digitalization for the Sustainability of the Healthcare System (DS3), Barcelona, Spain; 11 Servei de Farmacologia Clínica, Hospital de la Santa Creu i de Sant Pau, Barcelona, Spain; 12 Departament de Farmacologia, de Toxicología i de Terapèutica, Universitat Autònoma de Barcelona, Barcelona, Spain; 13 Laboratory of Molecular Neuropharmacology and Bioinformatics, Unitat de Bioestadística, Institut de Neurociències, Universitat Autònoma de Barcelona, Bellaterra, 08193, Spain; 14 Department of Psychiatry and Forensic Medicine, Medical School, Universitat Autònoma de Barcelona, Bellaterra (Cerdanyola del Vallès) Barcelona, 08193, Spain

**Keywords:** suicide prevention, suicidal behavior, mental health, real-world data, clinical decision support system, early warning system, electronic health records, AI, machine learning

## Abstract

**Background:**

Suicidal behavior is a major public health problem worldwide. The exact etiology remains unclear, representing a complex problem involving multiple factors. Evidence indicates that around 50% to 80% of people who die by suicide have had contact with the health care system in the year prior to their death.

**Objective:**

We present the IDICIUS project, whose objective is to develop a clinical decision support system that functions as an early warning system and applies AI to prevent suicide risk using anonymized electronic health record data.

**Methods:**

This study shows the first 2 stages of the IDICIUS project, where real-world data from 4 public sources were integrated, curated, standardized, and anonymized in phase 1, and analysis and modeling of suicide risk were performed in phase 2. This retrospective population-based study included 41,557 adult patients receiving mental health care at a large hospital. We evaluated the performance of machine learning classifiers with increasing complexity: logistic regression, elastic net, decision trees and random forests, bagging and boosting ensemble methods (GradientBoosting, XGBoost [extreme gradient boosting], CatBoost, RandomForest, and AdaBoost [adaptive boosting]), support vector machines, and deep neural networks. To address class imbalance, we tested several balancing techniques, with random undersampling providing the best results.

**Results:**

Phase 1 yielded a useful database of 32,661 patients receiving mental health care and 112 features. Of those, 2764 patients exhibited suicidal behavior (target), while 29,897 did not (control). The undersampling reduced the class distribution to 4422 vs 2211 (control vs target). The most prevalent features in the target class (N=2764) were psychiatric conditions, anxiety episodes (n=2269, 82.1%) and depressive episodes (n=1682, 60.9%), along with demographic and behavioral factors: female sex (n=1672, 60.5%), alcohol consumption (n=770, 27.9%), and conduct disorder (n=709, 25.7%). The largest patient subgroups were females with combined depression and anxiety (n=300, 10.9%), females with anxiety disorders only (n=182, 6.6%), males with anxiety disorders only (n=120, 4.3%), and males with combined depression and anxiety (n=106, 3.8%). In phase 2, ensemble methods, especially GradientBoosting and XGBoost, achieved the best performance, with receiver operating characteristic-area under the curve scores around 0.95. While these models showed moderate precision in identifying true positives (0.51‐0.58), they demonstrated high sensitivity in detecting at-risk patients, with recall scores of 0.85 and 0.84, respectively. Both models achieved a good balance between precision and recall (*F*_1_-scores: 0.68 and 0.67). The strongest predictors were corticosteroid use, psychiatric medications (olanzapine and lorazepam), female sex, and depression.

**Conclusions:**

We integrated multiple electronic health record data sources and applied AI to develop, model, and optimize ensemble algorithms for suicide prevention, maximizing effectiveness, efficiency, and generalizability. These AI-generated algorithms, based on readily available and well-structured data, may advance the identification of at-risk patients and function as early warning systems, thereby increasing opportunities for timely preventive interventions.

## Introduction

Suicidal behavior represents a complex problem with a substantial impact on public health worldwide [1-3]. Its heterogeneity and multifactorial nature make prediction in clinical practice particularly challenging, especially in the medium and long terms [4]. Nevertheless, evidence indicates that many individuals who die by suicide have sought some form of help prior to their death [5]. Approximately 50% to 80% of people who died by suicide had a prior interaction with the public health care system. Of these, around 60% did so during the previous month [6,7].

The implementation of early-stage interventions in this context is particularly crucial, as a prompt identification of individuals at high and imminent risk may allow for the effective mitigation of progression along the suicidal behavior continuum before it advances to more severe phases [8,9].

Although most prevention programs rely on training health care professionals to detect potential suicide risk and address it, recent reviews and meta-analyses suggest that such preventive measures may not achieve the expected effectiveness [10-14], and clinicians generally perform poorly at predicting who will attempt suicide [15]. This limitation highlights the need for novel approaches to identify patients at risk within health care settings—approaches designed not to replace clinical judgment but to complement and enhance existing assessment strategies [16].

In this context, electronic health records (EHRs) typically include longitudinal information, allowing for the monitoring of patients over extended periods and the identification of temporal patterns preceding suicidal behavior [17]. EHRs integrate a wide range of variables, including sociodemographic characteristics, clinical diagnoses, treatment histories, comorbidities, and health care utilization patterns [7]. Multiple studies have demonstrated that applying AI-based tools to EHR data can identify novel characteristics beyond the well-established risk factors for suicidal behavior [17-22], thereby enhancing the predictive accuracy of suicide risk models compared with previous approaches [17,23,24]. Furthermore, AI approaches using machine learning (ML) models enable the simultaneous assessment of multiple variables, capturing complex interdependencies [25,26] and nonlinear associations [27,28] in the development of predictive models.

The use of AI has also been extended to the development of early warning systems (EWSs) in hospitals as a clinical decision support system (CDSS) for clinicians [29,30]. These systems typically generate risk scores designed to assist clinicians in recognizing clinical deterioration among hospitalized patients. When integrated into EHRs, some EWSs have been associated with reductions in in-hospital mortality [31,32]. Building on this concept, similar AI-driven early warning approaches could be adapted to mental health care, enabling the timely identification of individuals at elevated risk for suicide and facilitating prompt, targeted interventions.

The IDICIUS project aims to develop a CDSS, motivated by EWS, that applies AI predictive models to prevent suicide risk. This tool will integrate information on suicide attempts, suicidal ideation, and deaths by suicide with clinical and sociodemographic factors derived from real-world data (RWD) recorded in EHRs and public health care and/or administrative databases, based on a cohort of 41,557 adult patients receiving psychiatric care. The IDICIUS project consists of a 4-stage approach, including (1) gathering of usable data, (2) analysis and modeling of risk, (3) building of a predictive tool, and (4) the implementation of the tool for effectiveness assessment. This study presents the first 2 stages of the IDICIUS project. Phase 1 focuses on integrating and harmonizing multiple databases with input from relevant stakeholders; implementing data curation, standardization, and anonymization; and defining key risk factors. Phase 2 aims to develop and validate predictive models using advanced ML techniques, selecting and optimizing the most effective, efficient, and generalizable algorithm for suicide prevention. Ultimately, these models are designed to assist clinicians in monitoring variations in suicide risk among patients and to be implemented within routine clinical workflows.

## Methods

### Sample Characteristics and Study Design

This is a retrospective, population-based study that used structured and anonymized existing EHR data from a large cohort of prevalent adult patients receiving mental health follow-up care in a large hospital facility (Consorci Corporació Sanitària Parc Taulí). The hospital provides outpatient care to a population of approximately 440,000 inhabitants in Sabadell, Catalonia. Predictive models were developed using AI algorithms and ML techniques, trained on the obtained data.

The study included data from cases who received mental health care between January 1, 2018, and June 1, 2024, at the hospital. The index visit was defined as the first recorded clinical encounter that qualified for study inclusion. To be eligible, patients were required to have had at least one additional visit within the 3 months preceding this index visit. Exclusion criteria included nonresidency in the Province of Barcelona and the absence of any visit to a primary care health center.

### Ethical Considerations

The study received approval from the institutional ethics review board under study code TED2021-131979B-C31 and case number 2023/5120. In addition, the study was approved by the institutional ethics committee to ensure compliance with the General Data Protection Regulation and applicable laws. As it used existing, fully anonymized data processed by an independent team, informed consent was waived by the ethics committee. Data confidentiality was maintained in accordance with Spanish Organic Law 3/2018 [33] and Regulation (EU) 2016/679 [34]. The study was conducted in accordance with the principles of the Declaration of Helsinki. The review process included 2 rounds of evaluation, with the initial submission on November 28, 2023, and final approval granted on January 25, 2024.

### RWD Sources

**Figure 1.**
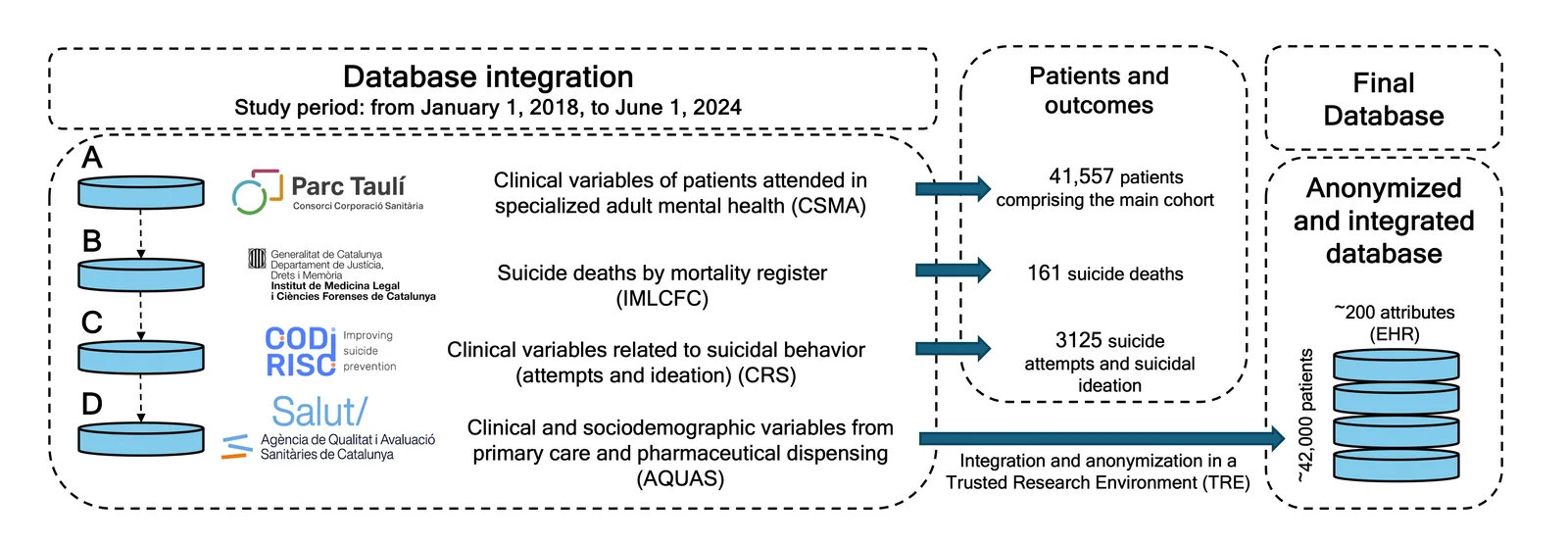
Real-world data integration schema. Four sources of information were integrated: (A) electronic health records (EHRs) registered from January 1, 2018, to June 1, 2024, at specialized mental health units for adults (Centre de Salut Mental per Adults [CSMA]) at Parc Taulí University Hospital (Consorci Corporació Sanitària Parc Taulí [CCSPT]); (B) deaths by suicide recorded by the forensic institute (Institut de Medicina Legal i Ciències Forenses de Catalunya [IMLCFC]); (C) clinical variables specifically related to suicidal behavior (Codi Risc Suïcidi [CRS]) registered by the Department of Health of Catalonia; and (D) clinical, sociodemographic variables, and pharmacy dispensation data registered by the Catalan Health Service (Catsalut) for invoicing purposes. All data were linked, anonymized, processed, and provided by AQUAS (Agència de Qualitat i Avaluació Sanitàries de Catalunya), a public-law entity under the Department of Health of Catalonia. After initial harmonization, data curation, and integration, performed at the Trusted Research Environment (TRE), a database of 41,557 patients and 206 features was obtained.

RWD was linked and extracted from 4 primary data sources (Figure 1):

1. EHRs of patients in mental health follow-up, attended at any level of care within the Parc Taulí Mental Health Service during the study period.
2. Catalonia Suicide Risk Code registry, containing sociodemographic and clinical information for individuals who have attempted suicide and/or present suicidal ideation assessed as high risk.
3. Registry of suicide deaths from the Institut de Medicina Legal i Ciències Forenses, the official source for identifying deaths by suicide.
4. Outpatient medication database of prescriptions billed by pharmacies to the Catalan Health Service, comprising all medications prescribed through the public health care system, dispensed by community pharmacies, and reimbursed.

Given the highly sensitive nature of the data, data linkage and extraction were performed by a team independent of the researchers, in accordance with legal requirements. The 4 data sources were subsequently harmonized, integrated, and anonymized by personnel from the Agency for Health Quality and Assessment of Catalonia (AQUAS [Agència de Qualitat i Avaluació Sanitàries de Catalunya]), a public-law entity under the Department of Health of the Generalitat of Catalonia holding the custody of large public health care databases for academic research. All data processing and modeling were conducted within AQUAS’s Trusted Research Environment, a secure computational infrastructure that prevents extraction of individual-level microdata under any circumstances and allows access to anonymized data only to authorized study personnel designated by the Department of Health of the Generalitat of Catalonia. Only aggregated statistical outputs were permitted for export, and these required explicit prior authorization through established data governance protocols.

### ML Classifiers and Hyperparameter Tuning

We evaluated the performance of state-of-the-art ML classifiers with an increasing degree of complexity: logistic regression, elastic net, decision trees and random forests, bagging and boosting ensemble methods (GradientBoosting, extreme gradient boosting [XGBoost], CatBoost, RandomForest, and adaptive boosting [AdaBoost]), support vector machines, and deep learning (DL). For the DL approach, we implemented a sequential neural network with 3 hidden layers (128, 64, and 8 units) using rectified linear unit activation, L2 regularization (0.001), and progressively adjusted dropout rates (0.3, 0.4, and 0.5). The output layer used a sigmoid activation for prediction. EarlyStopping and ReduceLROnPlateau were applied to prevent overfitting.

The optimal hyperparameters for the trained models were determined through grid search combined with 10-fold cross-validation, enabling systematic and robust tuning within the algorithms’ search space.

### Model Performance

Model performance on the target class was evaluated using standard performance metrics precision, recall, accuracy, and the *F*_1_-score, all of which are derived from true-positive (TP), true-negative (TN), false-positive (FP), and false-negative (FN) outcomes. Precision (TP/[TP + FP]) reflects how reliable the identified risk cases are; recall (TP/[TP + FN]) measures how many at-risk patients were successfully detected—that is, the ability to identify positives; accuracy represents the overall model correctness (TP + TN/[TP + FN + TN + FP]); and the *F*_1_-score combines precision and recall to measure how well the model balances correctly identifying patients who have the condition while avoiding misdiagnosing patients who do not, by using the harmonic mean: (2 × precision × recall)/(precision + recall).

In our clinical context, recall is the most critical metric, as an FN (failing to identify a patient at suicidal risk) can have severe consequences. Therefore, the model was designed to prioritize minimizing FNs, even at the expense of generating more FPs, which can subsequently be ruled out through clinical evaluation.

### Dataset Characteristics and Preparation

The integrated full dataset, combining the 4 RWD sources, consisted of 41,557 eligible patients with a total of 206 features. To construct the analysis dataset, one patient with a response rate below 5% was excluded, and the age range was restricted to 18 to 95 years to focus the study on the adult population, as risk factors significantly differ between adult and pediatric populations. The variable that registered the suicide attempts in the past 30 days (“count_temp_30d*”*) was also excluded to prevent data leakage with the target variable. The resulting analysis dataset comprised 32,661 patients and 112 features, ensuring data quality and relevance for subsequent analyses. Of those, 2764 patients exhibited suicidal behavior (target class), while 29,897 did not (control class).

Feature intersection analysis was performed using UpSet plots to visualize the complex overlapping patterns among features associated with the target class. This approach allows for the comprehensive examination of multiway intersections that extend beyond the limitations of traditional Venn diagrams. The analysis identified all possible combinations of these features and quantified the size of each intersection within the study population.

The analysis dataset was split into 80% training and 20% testing sets. To address class imbalance, we evaluated several balancing techniques, including SMOTE (Synthetic Minority Oversampling Technique), ADASYN (Adaptive Synthetic Sampling), SMOTE-Tomek, the Neighborhood Cleaning Rule, and RandomUnderSampler, both individually and in combination. Among these approaches, the highest performance was achieved using RandomUnderSampler alone. The RandomUnderSampler (sampler_strategy=0.5) reduced the class distribution from 23,917 vs 2211 to 4422 vs 2211 (negative vs positive cases). Prior to feeding the models, standard scaling was applied only to the numerical features (a total of 21).

To enhance model performance, we conducted additional feature engineering by applying log transformations to numerical variables and testing various mathematical transformations (divisions, sums, and logarithms) on key variables, including age, number of diagnoses, and medication count. However, these modifications did not improve the final model metrics.

### Explainability

We computed SHAP [35] (Shapley additive explanations) values for the classifiers to assess feature importance. The summary plot ranks features by influence (Y axis), while the X axis shows each feature’s impact on predictions. Positive SHAP values increase predicted suicide risk, whereas negative values decrease it, with color indicating high (red) or low (blue) feature values.

## Results

### Sample and Dataset Characteristics

The study included 41,557 cases and 206 features in the full dataset and 32,661 patients and 112 features in the analysis dataset. During the study period, 2604 individuals had a recorded suicide attempt and 160 died by suicide. In total, 2764 individuals had suicidal behavior and were defined as the target class (Table 1).

Among the target class (N=2764), the most prevalent features were psychiatric conditions, including anxiety episodes (n=2269, 82.1%) and depressive episodes (n=1682, 60.9%), followed by female sex (n=1672, 60.5%), alcohol consumption (n=770, 27.9%), and conduct disorder (n=709, 25.7%; Multimedia Appendix 1). The most common subgroup was females with both depression and anxiety (n=300, 10.9%), followed by females with anxiety disorders (n=182, 6.6%), males with anxiety disorders (n=120, 4.3%), and males with both depression and anxiety (n=106, 3.8%). In the analysis dataset, 60.5% (1672/2764) of suicidal behavior cases occurred in women; the prevalence of suicidal behavior was 9.0% (1672/32,662) in women and 7.7% (1092/32,662) in men.

**Table 1.** Sociodemographic characteristics and distribution of suicidal behavior.

Characteristic	Full dataset	Analysis dataset
Sample (n)	41,557	32,662
Age (y), mean (SD; range)	39.57 (22.19; 1-118)	47.08 (19.39; 18-95)
Sex distribution (total sample)		
Female, n (%)	22,150 (53.3)	18,518 (56.7)
Male, n (%)	19,407 (46.7)	14,144 (43.3)
Sex distribution by suicidal behavior		
No suicidal behavior, n (%)	38,432 (92.5)	29,898 (91.5)
Female, n (%)	20,182 (52.5)	16,846 (56.3)
Male, n (%)	18,250 (47.5)	13,052 (43.7)
Suicidal behavior, n (%)	3125 (7.5)	2764 (8.5)
Female, n (%)	1968 (63)	1672 (60.5)
Male, n (%)	1157 (37)	1092 (39.5)
Suicidal behavior rates by sex		
Female, n (%)	1968 (8.9)	1672 (9)
Male, n (%)	1157 (6)	1092 (7.7)

### Model Performance

**Figure 2.**
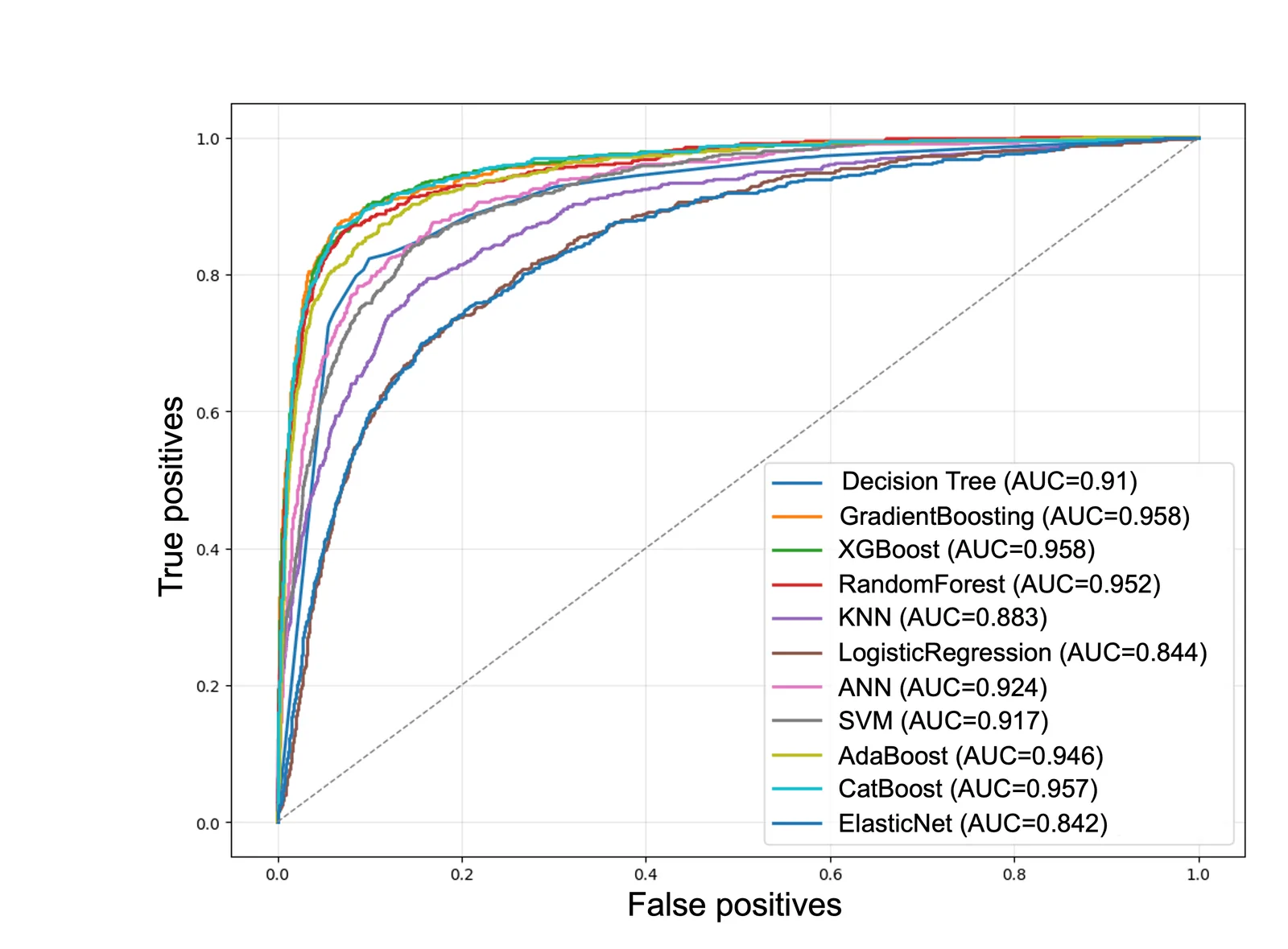
Receiver operating characteristic (ROC) curves for all models. ROC curves comparing the performance of 11 machine learning models for suicide risk prediction. GradientBoosting achieved the highest area under the curve (AUC; 0.958), followed closely by XGBoost (0.958) and CatBoost (0.957). Tree-based ensemble methods consistently outperformed traditional machine learning approaches, with LogisticRegression and ElasticNet showing the lowest performance (AUC=0.844 and 0.842, respectively). AdaBoost: adaptive boosting; ANN: artificial neural network; KNN: k-nearest neighbor; SVM: support vector machine; XGBoost: extreme gradient boosting.

Among the evaluated models, the ensemble methods GradientBoosting, XGBoost, CatBoost, RandomForest, and AdaBoost ranked highest, with receiver operating characteristic-area under the curve (ROC-AUC) values between 0.94 and 0.95 (Table 2 and Figure 2). These models also showed the highest accuracy, all above 0.91. Their precision ranged from 0.51 to 0.58, and recall ranged from 0.82 to 0.86. Support vector machine had the lowest precision (0.35) and recall of 0.81.

**Table 2.** Model performance metrics.

Model	ROC-AUC^a^	Accuracy	*F*_1_-score	Precision	Recall
GradientBoosting	0.9582	0.9343	0.6889	0.5751	0.8590
XGBoost^b^	0.9581	0.9313	0.6763	0.5624	0.8481
CatBoost	0.9574	0.9348	0.6872	0.5785	0.8463
RandomForest	0.9521	0.9300	0.6705	0.5576	0.8409
AdaBoost^c^	0.9458	0.9172	0.6256	0.5067	0.8174
ANN^d^	0.9241	0.9092	0.5850	0.4772	0.7559
SVM^e^	0.9165	0.8609	0.4975	0.3583	0.8137
DecisionTree	0.9104	0.8938	0.5673	0.4329	0.8228
KNN^f^	0.8826	0.9105	0.5193	0.4759	0.5714
LogisticRegression	0.8436	0.8054	0.3881	0.2644	0.7288
ElasticNet	0.8419	0.7964	0.3814	0.2567	0.7414

a ROC-AUC: receiver operating characteristic-area under the curve.

b XGBoost: extreme gradient boosting.

c AdaBoost: adaptive boosting.

d ANN: artificial neural network.

e SVM: support vector machine.

f KNN: k-nearest neighbor.

**Figure 3.**
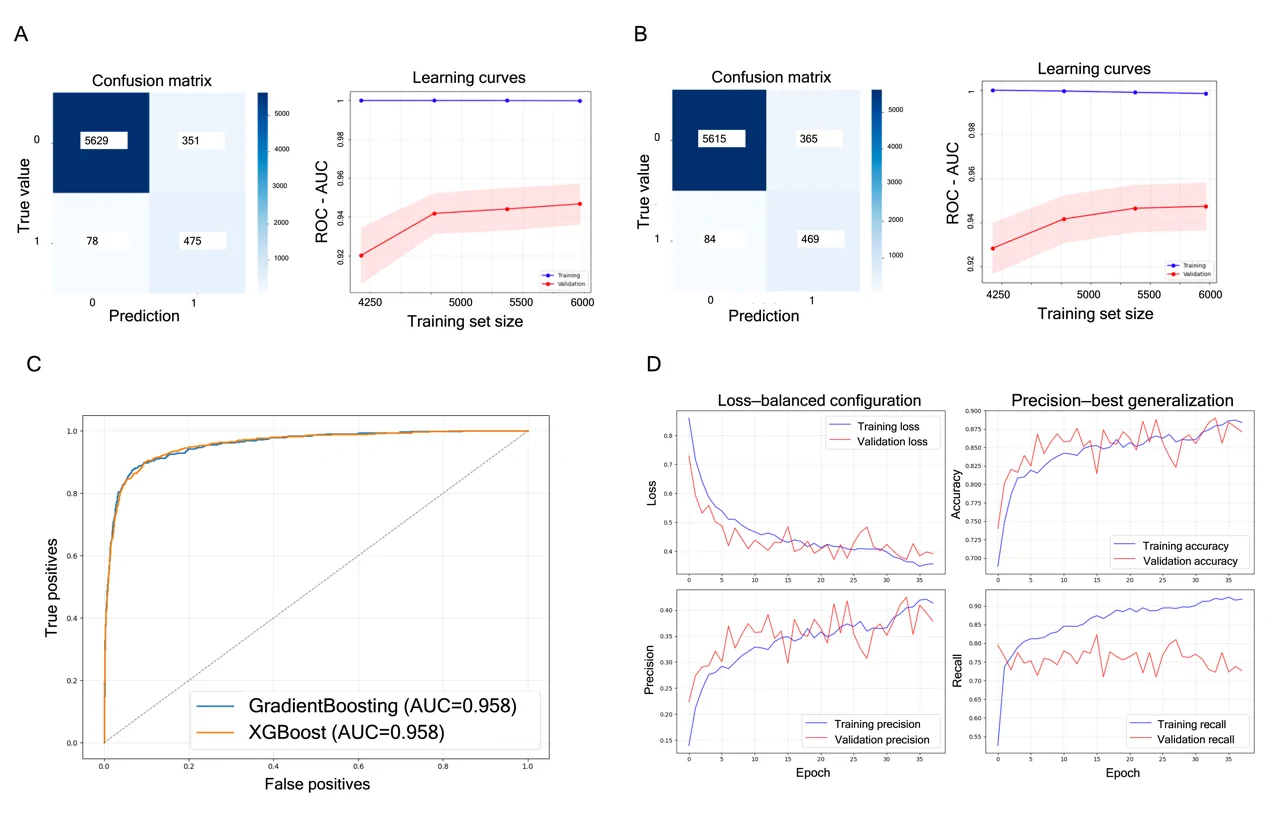
Performance analysis of top-performing ensemble models. Comprehensive evaluation of (A) GradientBoosting and (B) extreme gradient boosting (XGBoost) models showing confusion matrices and learning curves. The confusion matrices demonstrate high true-negative rates (5629/6533, 86.1% and 5615/6533, 85.9% for GradientBoosting and XGBoost, respectively) with relatively low false-positive and false-negative rates. Learning curves indicate stable performance across training set sizes with minimal overfitting. (C) Direct comparison of receiver operating characteristic (ROC) curves for both ensemble models demonstrates nearly identical performance with overlapping curves and identical area under the curve (AUC) values of 0.95. (D) Neural network training dynamics showing loss and performance metrics over epochs. The balanced configuration demonstrates stable convergence with training and validation losses decreasing consistently, while accuracy, precision, and recall metrics show steady improvement with minimal overfitting, indicating robust model generalization.

*F*_1_-scores varied across models, with the top-performing models ranging from 0.63 to 0.69. GradientBoosting achieved the highest *F*_1_-score (0.6889), followed by CatBoost (0.6872) and XGBoost (0.6763). Traditional statistical models performed worse across all 5 metrics. LogisticRegression and ElasticNet had ROC-AUC values below 0.85 and the lowest *F*_1_-scores (0.38 for both). KNN showed high accuracy (0.91) but lower recall (0.57). Overall, GradientBoosting and XGBoost were the best-performing models (Figure 3).

To test model stability and metric variability across imbalance-handling methods, we ran 1000 iterations of the best-performing model, GradientBoosting, under each strategy and summarized performance using mean estimates and 95% CIs (Table 3).

**Table 3.** Mean performance and 95% CIs of GradientBoosting across 1000 bootstrap resamples under different class-balancing methods.

Class-balancing method	ROC-AUC^a^, mean (95% CI)	Accuracy, mean (95% CI)	*F*_1_-score, mean (95% CI)	Precision, mean (95% CI)	Recall, mean (95% CI)
RandomUnderSampler	0.958 (0.946‐0.969)	0.934 (0.926‐0.942)	0.689 (0.648‐0.726)	0.575 (0.527‐0.622)	0.859 (0.816‐0.898)
SMOTE^b^	0.924 (0.907‐0.939)	0.919 (0.909‐0.928)	0.615 (0.570‐0.657)	0.514 (0.463‐0.566)	0.765 (0.714‐0.813)
ADASYN^c^	0.920 (0.903‐0.936)	0.921 (0.911‐0.929)	0.619 (0.577‐0.660)	0.523 (0.479‐0.569)	0.758 (0.709‐0.810)
SMOTE-Tomek	0.921 (0.904‐0.937)	0.917 (0.907‐0.926)	0.610 (0.567‐0.650)	0.506 (0.458‐0.552)	0.767 (0.717‐0.814)
Cost-sensitive learning (manual weights)	0.953 (0.936‐0.968)	0.958 (0.951‐0.965)	0.743 (0.69‐0.785)	0.782 (0.732‐0.828)	0.708 (0.654‐0.762)
Cost-sensitive learning (automatic weights)	0.952 (0.936‐0.967)	0.958 (0.951‐0.965)	0.742 (0.699‐0.784)	0.782 (0.726‐0.832)	0.707 (0.650‐0.758)

a ROC-AUC: receiver operating characteristic-area under the curve.

b SMOTE: Synthetic Minority Oversampling Technique.

c ADASYN: Adaptive Synthetic Sampling.

The neural network was evaluated with and without transformed numerical variables, with no relevant differences between the settings. Using the default threshold of 0.5, the model achieved an accuracy of 0.89, a precision of 0.43, a recall of 0.69, and an *F*_1_-score of 0.53. Threshold optimization identified 0.77 as the optimal cutoff, yielding a precision of 0.56, a recall of 0.58, and an *F*_1_-score of 0.57.

### Explainability

**Figure 4.**
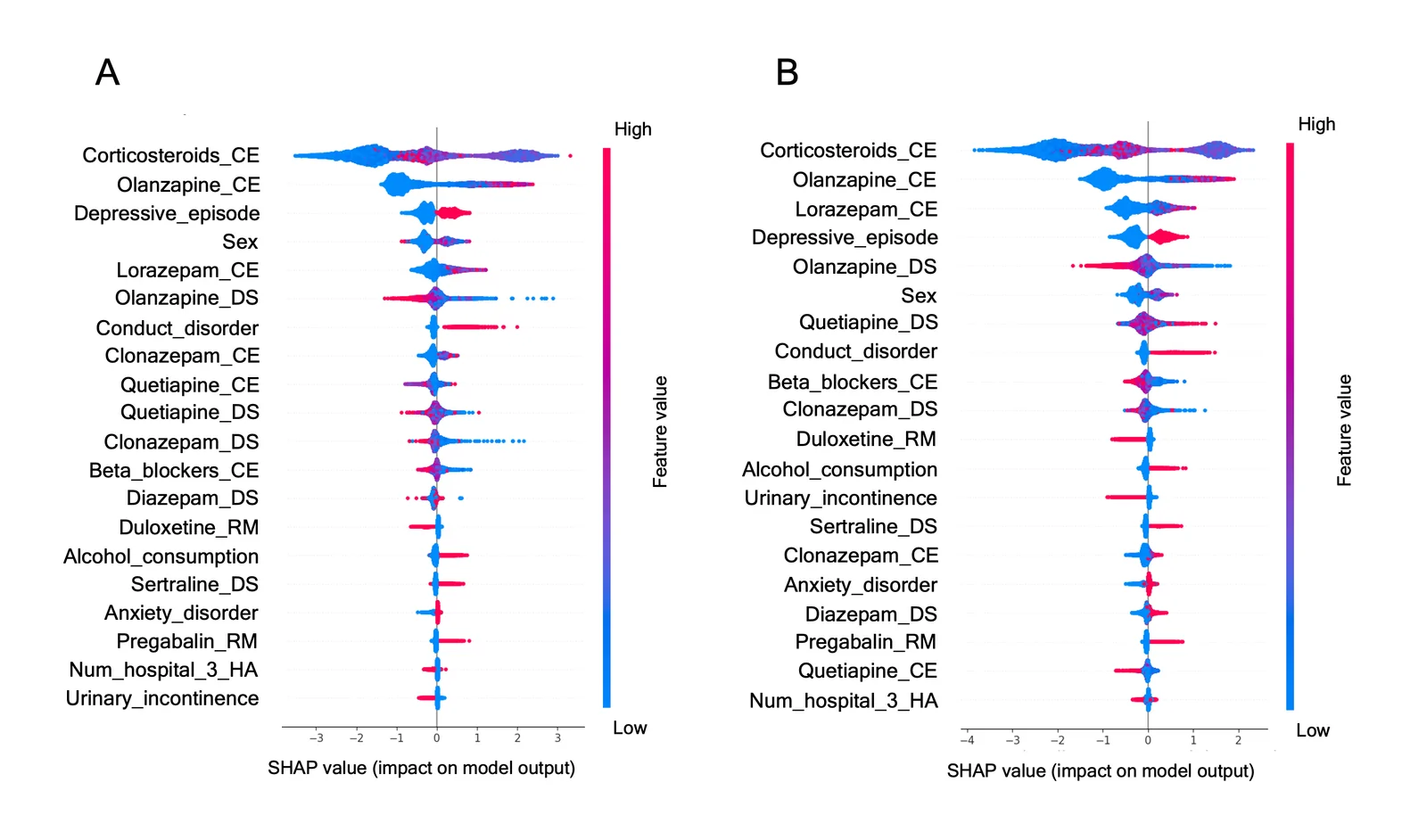
SHAP (Shapley additive explanations) values showing the impact of top features on suicide risk prediction for (A) GradientBoosting and (B) extreme gradient boosting (XGBoost) models. The consistent patterns between models validate the robustness of these feature importance rankings. Each point represents a patient, with the X axis indicating the feature’s contribution to the model’s output. Positive values increase suicide risk prediction, while negative values decrease it. Colors represent feature values (red color denotes “high” and blue color denotes “low”). Suffixes indicate drug intake timing: CE: current; DS: distant; RM: remote.

SHAP analysis showed similar feature-importance patterns for GradientBoosting and XGBoost (Figure 4). The most influential features included corticosteroid use, psychiatric medications such as olanzapine and lorazepam, female sex, and recurrent depression. Features such as urinary incontinence, hospitalizations, pregabalin use, and anxiety disorder were less influential.

## Discussion

This study evaluates the potential of AI for suicide prevention in a psychiatric setting using harmonized clinical data from 4 public databases. In a cohort of 32,661 patients receiving mental health care, suicidal behavior was associated with a relatively small set of common clinical and pharmacological features, including anxiety and depressive episodes, conduct disorders, corticosteroids, lorazepam, clonazepam, and olanzapine. Ensemble methods outperformed traditional approaches, in line with prior evidence that boosting methods often perform strongly across settings [36].

GradientBoosting and XGBoost showed the best overall performance (*F*_1_-scores of 0.68 and 0.67, respectively). In screening or triage settings, where FNs are particularly consequential, recall should be as high as possible. Here, recall was relatively high (0.85 and 0.84, respectively), supporting the potential use of these models alongside clinical judgment to guide targeted interventions. Accordingly, threshold selection should be guided by the clinical context and the relative costs of FPs and FNs [37]. A previous meta-analysis assessing ML performance in clinical populations reported a pooled recall of 0.58 [38]. Similarly, Pigoni et al [39] found substantial heterogeneity in recall/sensitivity across ML studies in psychiatric clinical populations, with values ranging from 0.13 to 0.98 and an overall mean of 0.72 after harmonizing percentages and range estimates. Traditional clinical judgment and standardized risk scales have demonstrated only modest predictive performance, slightly better than chance, although they may capture contextual and behavioral cues not reflected in structured data, such as affect or tone of voice [4,15]. At the same time, a substantial proportion of individuals who die by suicide have recent contact with health services, up to 80% within the year prior and 50% within the month before death [7]. Together, these findings highlight the potential value of data-driven approaches to support the identification of risk signals that may not be consistently detected during routine clinical assessment.

Precision was moderate (0.56‐0.57), indicating that some flagged individuals may not ultimately be at high risk. In the context of psychiatric follow-up, the additional burden of risk flags may be more manageable, particularly when alerts are restricted to patients already receiving care and when the output is used to support rather than replace clinical judgment. Although FPs can still contribute to unnecessary workload, the potential benefits of preventing suicide may justify a low-to-moderate positive predictive value when the intervention is cost-effective, actionable, and integrated into routine care. Indeed, comparable low positive predictive value strategies are routinely employed in other areas of health care, including breast cancer screening and stroke prevention [40]. Nevertheless, FPs should be minimized through threshold calibration, tiered risk stratification, and workflow integration.

Previous studies applying AI to EHRs have yielded mixed findings, with some reporting strong predictive power [27] and others showing good performance with classical statistical models such as LASSO or logistic regression [19]. Such variability likely reflects differences in methods, sample sizes, populations, and feature selection. Importantly, we excluded variables capturing prior suicide attempts, regardless of their known clinical value, to avoid data leakage. Although including these features markedly improved performance (precision from 0.56 to 0.98 for boosting methods), the models became overly dependent on them. Instead, we aimed to develop a clinically useful EWS that prioritizes recall and can identify individuals at risk even in the absence of prior attempts—when prevention is most challenging yet most critical.

Corticosteroid use consistently ranked as a highly important feature across both models, suggesting that it captures key clinical information, possibly reflecting chronic and severe underlying physical conditions linked to chronic suffering, underlying inflammatory conditions, or even a risk for corticosteroid-induced psychiatric symptoms and behavioral disturbances, and relapse or worsening of underlying psychiatric conditions [41]. Its prominence, alongside any other medications, may also indicate broader systemic issues. In GradientBoosting, corticosteroid use showed high variability in impact (SHAP values ~−2 to +3), while in XGBoost, its effect was similarly important but more constrained. Antipsychotic medications (olanzapine and quetiapine) and dosing patterns also contributed notably, potentially reflecting their role as markers for underlying severe psychiatric morbidity, such as schizophrenia, impulsivity disorders, or complicated depression, which are usual indications for their use and may be the actual underlying risk factors [7]. Similarly, clonazepam is often used for insomnia, a clinical condition that has been reported to increase the risk of suicide [42]. In these cases, exposure to these medications may be a useful marker of risk and is convenient for algorithms, since these are structured data that are readily available in databases [43]. Benzodiazepines, such as lorazepam and diazepam, ranked as a highly important feature across both models, are commonly used in psychiatric practice for anxiety, insomnia, and agitation [7]. The models incorporate demographic characteristics (eg, biological sex) alongside behavioral risk indicators (eg, alcohol consumption). Together, these clinically familiar features provide face validity to the model and integrate multiple variables simultaneously rather than relying on any single risk factor, potentially highlighting risk patterns that may not be readily apparent in routine practice. Thus, value is added by ensuring completeness in recovering and summarizing relevant risk information from existing records, easing the health care professional’s task. This may be particularly relevant in primary care and emergency settings, where EHRs can be incomplete, fragmented across care environments, or not immediately accessible and where timely risk identification and stratification are essential.

While EHRs provide valuable clinical information, they fail to capture the full multidimensional complexity of individuals’ lives. We previously reported [44] that DL models improved the detection of TPs by incorporating domains absent from EHRs, such as risk-taking behavior, social media activity, social connectedness, sexual behavior, and personality traits. In that study, recall ranged from 0.67 to 0.87 and accuracy from 0.83 to 0.87, although precision remained lower. Subsequent work further improved performance, with the best models achieving area under the curve values of 0.85 to 0.90 and recall values of 0.81 to 0.93, but with very low precision of 0.17 to 0.16. These results highlight that suicide risk prediction may benefit from integrating routinely collected clinical data with broader psychosocial and behavioral dimensions.

However, ensuring responsible data integration requires coordinated efforts across stakeholders while maintaining privacy and ethical standards. In addition, generalizability remains a major challenge, as models developed in one population may not transfer well to others due to differences in ethnicity, age, cultural context, or clinical characteristics [39,45,46]. Furthermore, methodological variability in EHR recording can introduce additional inconsistencies, even across hospitals within the same country [47]. For instance, our control group consisted of individuals receiving psychiatric treatment, reflecting a clinically realistic population in which distinctions between individuals at varying levels of suicide risk may be more subtle.

Suicide is a global public health problem, accounting for 1.5% of all deaths worldwide [3,46,48]. Public health action is urgently needed, and EHRs appear well-suited for developing suicide prevention algorithms and CDSS, as they can capture and integrate newly available clinical information in real time to help prevent deaths. The system is intended to support, not replace, clinical judgment, and its value will depend on integration into existing care pathways. In this setting, FNs are the more consequential error because missed cases may delay or prevent timely intervention, whereas FPs can be addressed through confirmatory assessments and structured follow-ups. Once a risk flag is generated, the model could trigger evidence-based responses such as safety planning-type interventions, associated with a 43% reduction in reattempts over 12 months [49], and brief contact and continuity interventions, which have been shown to significantly reduce suicide reattempts [50]. The Catalan Suicide Risk Code provides a real-world example of this integrated pathway, linking detection with structured follow-up and continuity of care [51]. Implementation should also include ethical safeguards such as subgroup monitoring, patient transparency, and institutional governance.

Key limitations include restricted generalizability, a lack of broader psychosocial data, and a relatively small sample size reduced further by undersampling. To improve generalizability, we applied techniques such as cross-validation, separate training and testing sets, and bootstrapping. These limitations will be partially mitigated through clinical validation and large-scale implementation in the public health system, enabling evaluation in large populations. The clinical validation of these findings is a critical next step, planned as part of the second phase of this project. This will involve implementing the algorithm within the hospital information system and prospectively validating its predictions against newly collected clinical data. While we removed variables known to improve metrics, such as prior suicide attempts, with the aim of predicting nonobvious risk of suicidal behavior, known risk factors may be used in combination with the proposed algorithms to complement the detection of individuals at unknown and known risks. In summary, the use of AI-generated algorithms based on readily available and well-structured data may allow us to make progress in the identification of individuals at risk, thus increasing the chances of applying preventive measures in a timely manner.

## Supplementary material

10.2196/85085 Multimedia Appendix 1 UpSet plot showing the distribution and overlaps of 10 key clinical variables.

## Abbreviations

AdaBoost: adaptive boosting
ADASYN: Adaptive Synthetic Sampling
AQUAS: Agency for Health Quality and Assessment of Catalonia
CDSS: clinical decision support system
DL: deep learning
EHR: electronic health record
EWS: early warning system
FN: false-negative
FP: false-positive
ML: machine learning
ROC-AUC: receiver operating characteristic-area under the curve
RWD: real-world data
SHAP: Shapley additive explanations
SMOTE: Synthetic Minority Oversampling Technique
TN: true-negative
TP: true-positive
XGBoost: extreme gradient boosting
