# Supplementary material for: AI Models for Suicide Risk Prediction in Adult Patients Receiving Mental Health Care Using Real-World Data: Retrospective Population-Based Study
Source: JMIR AI. 2026 Aug 26;5:e85085. doi: 10.2196/85085 (PMC13515357; doi:10.2196/85085)
Supplement: Supplementary file 1 [file ai-v5-e85085-s001.docx]

Figure S1. UpSet plot showing the distribution and overlaps of key clinical variables. The horizontal bar chart (left) displays the frequency of each individual feature, while the vertical bar chart (top) shows intersection sizes with corresponding percentages. Connected dots in the matrix indicate which features are included in each intersection. Psychiatric conditions, anxiety episodes (82.1%) and depressive episodes (60.9%) dominates most of the intersections.
